# An agenda for addressing bias in conflict data

**DOI:** 10.1038/s41597-022-01705-8

**Published:** 2022-09-30

**Authors:** Erin Miller, Roudabeh Kishi, Clionadh Raleigh, Caitriona Dowd

**Affiliations:** 1 Global Terrorism Database (GTD) START, University of Maryland, Box 266, 5245 Greenbelt Rd, College Park, MD 20740 USA; 2 Armed Conflict Location & Event Data Project (ACLED), 361 Falls Rd. #501, Grafton, WI 53024 USA; 3 Armed Conflict Location & Event Data Project (ACLED),, ACLED and University of Sussex, Brighton, BN1 9RH United Kingdom; 4 Dublin City University Collins Ave Ext, Whitehall, Dublin, Ireland

**Keywords:** Politics, Research data

## Abstract

With increased availability of disaggregated conflict event data for analysis, there are new and old concerns about bias. All data have biases, which we define as an inclination, prejudice, or directionality to information. In conflict data, there are often perceptions of damaging bias, and skepticism can emanate from several areas, including confidence in whether data collection procedures create systematic omissions, inflations, or misrepresentations. As curators and analysts of large, popular data projects, we are uniquely aware of biases that are present when collecting and using event data. We contend that it is necessary to advance an open and honest discussion about the responsibilities of all stakeholders in the data ecosystem – collectors, researchers, and those interpreting and applying findings – to thoughtfully and transparently reflect on those biases; use data in good faith; and acknowledge limitations. We therefore posit an agenda for data responsibility considering its collection and critical interpretation.

## Introduction

With increased availability of disaggregated conflict event data for analysis, there are new and old concerns about bias. All data have biases, which we define as a systematic inclination, prejudice, or directionality to information. Bias occurs when a dataset deviates from a pure/comprehensive model of reality in non-random ways that may produce misleading and harmful inferences if not accounted for properly. In conflict data, skepticism about potentially damaging biases can cause doubt about whether data collection procedures create systematic omissions, inflations, or misrepresentations due to the aforementioned prejudices or directionality. As curators and analysts of multiple large, popular data projects, we are uniquely aware of biases that are present when collecting and using event data. We have observed that researchers can significantly misinterpret the effects of biases, both overstating and understating the significance of certain biases. We contend that it is necessary to advance a more nuanced discussion that goes beyond the question of whether biases exist, and articulate the responsibilities of everyone in the data ecosystem – collectors, researchers, and those interpreting and applying findings – to more thoughtfully reflect on potential biases, use data in good faith, and acknowledge limitations of data collection and critical interpretation.

Systematic biases in conflict datasets take many forms, both intentional and unintentional. Some biases arise from challenges surrounding access to information or popular attention to certain issues. Perhaps more significantly, biases also emerge from numerous strategic decisions dataset creators make regarding the types of conflict data collected and methods of information sourcing, to minimize errors, maximize curation of the highest quality information, and align with the project’s goals. These decisions, which balance trade-offs and tensions in intention versus abilities, largely determine the size and shape of conflict datasets. There are no ‘right answers’ that accommodate every research scenario, but unintended biases and intentional strategic decisions alike should be documented and carefully considered for their particular relevance to contexts, actors, and research questions. To promote a more nuanced discussion of bias as it relates to the complexities of conflict environments and data collection methods, we first review instances of bias arising from error, then turn to the implications of decisions and strategies of data collection efforts, before lastly identifying responsibilities of different roles in the research cycle. Our goal is not to compare specific datasets to further a debate about their respective merits, but instead to recognize that datasets frequently differ for both intentional and unintentional reasons. To effectively build knowledge, all involved in the research enterprise must aim to understand the causes and effects of these differences, and the trade-offs they present.

## Bias as Error

We focus on three key categories of conflict data error: (1) omission; (2) inflation; and (3) misrepresentation. Errors of this kind do not only result from biases (systematic inclinations, prejudices, and directionality in information), but can also in turn contribute to further biases. Specific strategies can effectively prevent and reduce many errors of these kinds during data collection and review processes.

### Omission

Omission errors are those resulting from insufficient attention to or coverage of types, places, or periods of conflict that a dataset aims to cover. For example, if a dataset claims to cover violence against civilians by armed, organized groups, but neither defines nor sources those events uniformly and comprehensively, errors of omission occur. No dataset can claim to comprehensively capture the true ‘universe’ of all violence which occurs, and random errors should not affect overall analysis, but systematically excluding a significant amount of relevant information can have deleterious effects.

Common types of omission that users presume are systematic include geographic disparities in coverage. Whether urban events or rural events are adequately or fully covered is partially due to assumptions about sourcing information, and how rural events are not reported as well or as thoroughly as urban events^1^. There are political and contextual reasons why some rural events are under-reported and possibly omitted. While events from national or regional sources are reported in widely-spoken languages and are accessible to most data collection programs, rural information is typically more inaccessible due to local, non-English languages. Rural events may be solely reported via radio broadcasts; online in sub-national media; or collected through local organizations. The investment in research and journalism required to collect and translate information across multiple types of conflict environments is significant. Data projects that make these investments will have lower levels of omission biases compared to those that do not.

Despite these investments, not every event will be captured as not every event is reported and even fewer can be verified. Random cases of omission may have little influence on research as there is no ‘true’ catalog of all violence. However, systematic omission and omission that varies due to time-sensitive, conflict-related events can be an enduring source of bias that has only partial solutions. In some contexts, there may be poor reporting in a closed media environment due to state suppression of journalism (e.g., Eritrea; North Korea) while elsewhere, highly partisan sources may systematically exclude reports of violence by aligned political actors. Improving access to information can help but is not always possible and creates new challenges of uneven coverage over time and place.

### Inflation

Data analysts often focus exclusively on concerns related to under- or non-reporting. Meanwhile, data creators are faced with clear areas of excessive reports (e.g., duplicate reports; erroneous inclusion of non-events). There is often a presumption that more data are better data, which can create incentives for inflation.

Projects that rely exclusively on artificial intelligence and ‘big data’ to produce structured event data from unstructured text have received significant attention. If taken at face value, the size of these collections may give the impression that a vast number of conflict events are missed by researcher-led datasets due to the comparatively limited capacity of subject-matter experts. Even when those presumptions are proven false^2^ and the large tranche of false positives are uncovered^3,4^, many analysts believe that automated data collection provides a relatively accurate ‘big picture’ of patterns, while getting some smaller details wrong^5,6^.

However, despite relative advantages in the speed and volume of data collection, automated approaches are vulnerable to trade-offs in the accuracy of the data collected, often encompassing errors in geographies, actors, timelines, and targets^7^. They are frequently noisy with false signals and patterns, specifically because of poor scope and catchment boundaries, data inflation, and excessive duplication. Especially egregious errors may introduce ‘conflicts’ where none exist, through the creation of numerous records based on source documents describing fictional accounts, opinions, marketing campaigns, entertainment, or pop culture that adopt terminology associated with conflict – e.g., a verbal *attack* or a film that *bombed* at the box office. Date resolution errors may cause a high-profile historical attack to re-appear in a database due to media coverage on an anniversary years later. False records and actual conflict events are sometimes duplicated many times over due to multiple reporters covering the same event, or translation and syndication of identical reports. These issues are rampant across automated systems^8^, and are particularly pronounced in relation to specific event characteristics – like fatalities – which are vulnerable to politicization by different parties^9,10^. Researcher-led datasets address these threats by putting in place safeguards, such as clear definitions; a selection of valid, reliable, and relevant sources; and review processes conducted by experienced personnel capable of reading comprehension and disambiguation.

### Misrepresentation

A serious issue with any information collection is the presence of misinformation, disinformation, propaganda, and partisan (mis)characterization of events. Data collectors’ internal procedures can stem the effects of much untrue information, including triangulating information where possible; carefully choosing and interrogating source intentions and biases, including local organizations and their remits; considering the ‘mistake rate’ of sources and what kinds of information are more likely to be misrepresented; and limiting the use of social media to confirmed and vetted sources. Multiple independent sources may be used to draw out information for a single event, and propaganda can acknowledge an event took place without establishing its details. Experienced data collectors routinely adopt multiple strategies in a continuous effort to guard against inaccuracies caused by biased source materials, while maximizing the amount of reliable information available.

Some adopt an oversimplified view of this challenge, and both producers and users of data may act in bad faith when assessing and addressing concerns about partisan bias. There are obvious signs when this is true: poor methodologies; insufficient attention to sourcing information to mitigate biases; and the presence of partisan rhetoric, analysis, and framing are clear examples. However, it is also problematic to conflate partisan bias in sources with bias in the resulting dataset. For example, consider concerns that the inconsistent use of the word ‘terrorism’ by authorities and journalists describing similar events indicates insidious prejudice based on the race, ethnicity, or religion of the assailants or targets of an attack. This is a serious and valid critique that demands attention. However, a rigorous data collection initiative will adopt standardized definitions that are independently assessed based on the underlying characteristics of the attack, rather than labels applied by third parties. Failing to understand or acknowledge the procedures in place to prevent partisan framing from compromising the objectivity of a dataset may lead an observer to overstate this particular risk, while understating less visible challenges which may or may not be partisan, such as threats to the security of journalists or observers reporting on conflicts.

## Underestimating the Impact of Dataset Design Decisions

Many critics focus on unintended or partisan systematic errors as sources of bias, but overlook how strategic decisions about the design of data collection strongly influence which conflicts are captured and the profile of violence this represents. We focus here on three foundational decisions regarding: (1) responding to conflict dynamics; (2) prioritizing stability or comprehensiveness in data sourcing; and (3) source information breadth and depth.

Datasets measuring conflict prioritize the constituent events of political violence differently, leading some to emphasize threats to government sovereignty, others to focus on ‘terrorism’, or to capture a wider spectrum of political disorder. Many data collections deliberately represent specific forms of conflict, rather than all forms. How they do so has important implications for the resulting data and their use.

### Responding to evolving conflict dynamics

The nature of conflict is changing and violent environments evolve over time. For example, a conflict may escalate from civil war to regionalized conflict or shift from being dominated by civil war violence to lower intensity political violence involving a more diverse range of political and criminal actors. A dataset can either adapt when conflicts shift, to capture the dynamics of insecurity as they unfold, or not, adhering to established definitions and thresholds.

There is an inherent trade-off in prioritizing ‘reliability’ over ‘validity’ in information collection. *Reliability* refers to consistency and stability in data collection over time, maintaining the same definitions, thresholds, and boundaries. Such constancy allows for comparisons between similar events over time and place based on a consistent set of definitional and conceptual parameters. However, this comes at the cost of potentially missing the complexity of evolving conflict environments that transform beyond definitional and conceptual boundaries as originally envisaged. As new and previously unrecognized forms of violence emerge, an alternative approach is to prioritize the *validity* of data collection, or the extent to which a measure accurately captures what it is intended to.

For example, recent decades have witnessed profound shifts in how security is defined, conceptualized, maintained, and undermined beyond and below the level of the state. This has included greater consideration of violence once neglected or ignored entirely in conflict scholarship, such as that perpetrated by irregular, non-state forces; paramilitaries; and militias. These groups are not uniquely modern phenomena, but they have only relatively recently been recognized by mainstream peace and conflict studies as prominent actors across a range of conflict environments. As broader conceptual debates shift the parameters of what scholarly, policy and security actors recognize as relevant forms of violence, persistent adherence to older definitions and parameters, to the exclusion of such acts or actors, may itself constitute a form of ‘conceptual bias’. This is particularly the case for data collection efforts explicitly aimed at capturing broad patterns of violence. For datasets with this mandate, accounting for the evolution of conflict (and our understanding of it) over time will be a priority, while collections with a narrower remit and mandate – for example, to consistently capture a specific form of violence – may instead adhere to established decisions about the inclusion and exclusion of particular acts. Both approaches are valid with respect to the phenomena for which they are designed, but have vastly different strengths and limitations for analysis.

Depending on the goals of data collection and the research questions at hand, there are trade-offs between reliability and validity. Effectively, when do the changing parameters of violence create systematic exclusions in conflict datasets? At one end of the spectrum, rigid definitions can make conflicts ‘disappear’ by excluding information; at the other end, an overly fluid catchment can undermine comparisons or ‘create’ conflicts through inconsistent inclusion. The most significant problems arise when the collection’s stated agenda, analytical objectives, and approach are at odds – e.g., a project purports to cover a wide spectrum of conflict, but chooses a methodological approach that systematically excludes relevant events, resulting in a narrow (biased) range. Likewise, a project designed to capture a particular type of conflict cannot be used to understand evolving types of conflict events that are out of its scope. There is no single ideal approach, but these trade-offs highlight the need for more explicit recognition of data collection objectives and decisions across contexts.

### Responding to evolving dynamics of sourcing

The changing information environment has similar implications for reliability and validity. Reliability in sourcing refers to consistency and stability in the sources used over time. To ensure stability, easily and consistently accessible sources may be prioritized — often meaning international, English-language sources. Such constancy facilitates comparisons and modeling over time and place. However, this stability may be an illusion: strong internal consistency of reporting by major international outlets cannot be assumed, and it can come at the cost of missing the complexity of conflict environments.

Sourcing validity refers to the extent that sources accurately capture the conflict trends in question. In this regard, data collection must strive to bring together the most effective constellation of sources in each context, together most accurately capturing events. Changes in the availability of sources are inevitable and may be produced by two different scenarios. First, sourcing changes may be endogenous to the conflict itself as shifts in the political landscape impact conflict trends, which in turn shape the reporting efficacy of sources. In this scenario, a sourcing strategy that prioritizes validity needs to adapt to changes in a conflict environment if it is to accurately capture evolving trends, despite threats to the stability of sourcing.

A second scenario is that changes in the availability of sources occur independently of conflict patterns. The changing salience of particular types of violence or terminology used to describe violence can correspond to shifts in the resources dedicated to reporting, collection, and analysis of those types. These, in turn, shape the extent to which certain forms of violence are captured in datasets, without necessarily reflecting a shift in underlying patterns of violence. For example, the gradual recognition of conflict-related sexual violence as a specific and strategic tactic in conflict has fueled demands for data which accurately quantify and track it over time and place^11^. Increased records of conflict-related sexual violence may be a function of the increased political and policy salience of the issue, and more comprehensive coverage as a result.

Ultimately, data collectors have a choice to make between adhering to consistent sourcing or dynamically adapting their strategy over time. Adding new sources to coverage introduces certain biases and potential distortions impacting temporal analysis, including the intensity and frequency of conflicts^12^. Our argument is not that a dynamic sourcing approach is the optimal method for all data collection and resulting analyses, but rather, to draw attention to the lesser-recognized fact that *not* adding new sources and methods to coverage also entails trade-offs. Adhering to rigid sourcing introduces *other* biases, as reported conflicts are certainly missed. This results in an inaccurate picture of the conflict environment, particularly for those collection efforts with a stated objective of capturing a wide range of political violence.

Again, the impact of these trade-offs depends in part on the objectives of the researchers. Static and focused sourcing, rather than dynamic and investigative ways to approach changing information environments, is particularly damaging because they will likely result in under-representation, distorted trends, and incorrect conclusions. Data projects that do leverage a variety of sources — international, regional, and local; English and non-English — must make significant efforts to monitor access to information, adapting as platforms come and go and reporting environments evolve in response to shifting conditions. However, in doing so, there must also be consistency in the overall strategy used to mitigate biases and to avoid the distortion of conflict patterns, introducing artificial spikes or dips in trends in response to rapid changes. Sources should be added or subtracted with careful consideration. Balancing these many facets is a delicate act, yet imperative for analysts to understand.

### Breadth and depth of source information

The quality of all conflict data rests on its sourcing and information management; most collections aggregate a variety of source types. Each source type provides certain strengths that contributes to establishing a complementary picture of conflict trends in specific environments. Additionally, each source type also comes with certain limitations to be considered.

For example, studies of traditional and ‘new’ media suggest that, *in combination*, they are complementary, and cover much of the actively reported events in crisis environments. Reports from ‘new media’ may be “more geographically concentrated, particularly in the capital city and wealthier areas”, and more timely around crisis contexts such as elections, while traditional media reporting tends to have “a wider geographic reach” with more consistency outside of immediate crisis periods, such as in the lead up to and aftermath of elections^13^. Further, national media may be integral in one context with robust and free press, yet local conflict observatories and ‘new media’ may play a more important role in a more closed press environment.

Neither the number of sources, nor specific types of sources, will universally guarantee the quality of data. International media are generally less vulnerable to partisan influences and are more accessible, but typically report on major events or those in urban contexts, while local media focus more on smaller events, and in more rural contexts. Adding *more* international media is unlikely to fully resolve existing biases — it will simply introduce more reporting around the same major events. Instead of simply increasing the number of sources used, data creators should identify which source constellations maximize event coverage while mitigating reporting biases, as appropriate for the types of events in question.

There are inherent biases in choosing one scale of information over another as the broader narratives of conflict are differently emphasized. International media often track high-profile events of interest to a broad audience, rather than a full accounting of all conflict activity. These media tend to frame conflicts for a primarily international audience in their reporting — leading to, for example, contexts with certain prejudices or with more editorial coverage^14^. They are also heavily skewed towards reporting in English, and provided by foreign correspondents often based in capital cities without access to all parts of a country. These practices exacerbate urban bias.

Collecting accurate information through different sources is beset by risks and barriers. Accurate data collection is about constructing a puzzle from many disparate pieces, each contributing partial information of the greater whole. For example, incorporating local partner information can involve trade-offs in how consistently information is conveyed. Local media can also introduce biases, and its information still requires careful review, as editorial distortions can be introduced by political, state, or ideological positions: potential biases from local media and reporters have been well-documented^15,16^. Ultimately, the trade-offs inherent in sourcing decisions and the relatively common tendency to emphasize consistent, international, English-language source selection at the expense of customized sourcing strategies, should be examined as part of a wider reflection on bias and its implications in conflict data. As above, the significance of these trade-offs will vary according to the specific and stated mandate of the data collection initiative itself, with the resulting exclusions and biases weighed accordingly.

## An Agenda for Responsibility

Analysts often recognize that biases exist and seek to correct them using analytical strategies or techniques. Thoughtful use of such strategies may help mitigate the effects of bias. For example, measures that are less precise may be less vulnerable to bias. A binary measure of whether a location experienced violent conflict in a particular year is more robust than a specific number of attacks; categorical data on casualties is more reliable than precise numbers of those killed or injured; and aggregating data spatially or temporally may be wise, depending on one’s research question.

Likewise, sophisticated modeling techniques such as statistically controlling for time dependence, using instrumental variables to account for measurement error and endogeneity, or relying on automated techniques to combat ‘missingness’ are appealing and may improve certain issues if used thoughtfully. However, they do not absolve the analyst of responsibility to engage with the substantive implications of measurement challenges or conceptual choices of the data project. On the contrary, ensuring that control variables are specified and interpreted properly, and justifying the use of instrumental variables that satisfy the necessary assumptions for validity, are critical yet often overlooked tasks^17,18^. Moreover, when empirical strategies are adopted as a substitute for confronting the substantive political realities that generate and perpetuate biased information in conflict environments, they can provide a false sense of security and be quite detrimental. Worse still can be when such efforts introduce ethical consequences: when precision and recall metrics are poor, they can have inadvertent implications, such as misidentified perpetrators of atrocities, as an example.

Efforts to support the integration of conflict datasets with other sources of information can be useful by providing access to multiple open-source data collections in one electronic archive, while preserving the decisions data collectors made in developing their projects. xSub, for example, brings together disparate sources to one place as a repository^19^. When combining different data frameworks, analysts must consider the implications of aggregation choices, including spatial and temporal measures or event/actor typologies. These decisions can introduce additional sources of bias and compromise results, so they should be handled with care. Endeavors that document how data collection frameworks can be reasonably aligned, but also acknowledge the ways they are fundamentally incompatible, are constructive.

Other efforts, however, especially those that strive to integrate datasets for the purpose of correcting perceived deficits, can be more problematic. Attempts to generalize and combine independently produced datasets have little regard for purposeful differences in data collection methodologies or the limitations of automated de-duplication, and hence risk creating uninterpretable ‘Franken-datasets’. While such efforts may be attractive to analysts wishing to conduct robustness checks, a more appropriate strategy for checking the robustness of research results might be to conduct independent analyses, maintaining the integrity of the original datasets in order to qualitatively assess the substantive differences. If a sufficient body of literature exists on a particular topic, a meta-analysis might be informative.

What is required, therefore, is greater recognition of the diverse factors that contribute to information bias in conflict environments; the intentional and unintentional data collection strategies that can either mitigate or exacerbate these; and the subsequent implications and trade-offs of design choices of different data collection efforts. Research questions and applications are unequally affected by the unintended biases found in any dataset, as well as by the intentional decisions of conflict event dataset creators. Ultimately, biases are far more complex than anticipated. They are not unidirectional, as multiple, co-occurring forms of bias often function in countervailing ways to reinforce and mitigate one another. Nor are they uniform across contexts or time: conflict contexts and the nature, extent, and impact of biases shift and evolve. Lastly, biases are not simply a function of low-information environments. Neither a larger volume of information, nor greater dependence on sources at a certain scale can guarantee unbiased data.

Taken together, the conditions outlined above highlight the value of more nuanced approaches to identifying, assessing, and mitigating bias in conflict data collection, analysis, and interpretation than has characterized conflict research to date. They also point to the various responsibilities that fall on data collectors, researchers, and those who analyze and interpret data, which we outline below.

Data collectors have responsibilities to clearly and explicitly articulate the collection mandate and decisions of their project, and delineate its parameters of inclusion and exclusion. They must design data sourcing strategies that credibly align with their stated agenda and must develop data collection protocols that capture relevant events within these parameters as rigorously, comprehensively, and systematically as possible. They should employ stress-testing strategies for systematic exclusions and missing events, and should develop and apply appropriate mitigation measures accordingly. Periodically, they should interrogate the dynamic evolution of conflict environments and the implications this has for the trade-offs between stability and accuracy of strategies and sources. Data collectors must explicitly acknowledge limitations, caveats, and gaps where, even with mitigation, known and suspected biases that undermine the stated parameters persist.

Researchers using these resulting data also have challenging responsibilities. First, they must select appropriate datasets for analysis with due consideration of the stated aims and agenda of the data project and its inclusion and exclusion parameters, assessing the implications for their particular analytical objectives in good faith. This means honestly evaluating the suitability of a dataset, without underestimating *or* overstating a dataset’s biases or limitations. Independent, critical assessments of datasets are absolutely essential, but those unfamiliar with a particular project’s methods must avoid misrepresenting or propagating inaccurate assumptions. That said, researchers should consider the potential for biases — both known and suspected — to undermine the specific focus of their intended research, even in ways not anticipated by data collectors. Analysts must also interrogate the trade-offs between reliability and validity regarding definitions and sourcing, and in light of their specific research question, revise accordingly. Where possible, analysts should introduce statistical and analytical techniques to account for and minimize known and suspected biases that undermine the suitability of the dataset for their specific research question. Presenting results thoughtfully, and accurately acknowledging limitations and caveats due to potential biases that cannot be mitigated, is crucial.

Lastly, those interpreting and applying the findings of research drawing on conflict data bear responsibility for assessing and contextualizing those findings. This includes selecting appropriate research findings for discussion in relation to particular conflict environments, and critically evaluating the relevance of potential biases that might undermine the interpretation of findings in particular contexts.
